# Insights on the Influence of Surface Chemistry and Rim Zone Microstructure of 42CrMo4 on the Efficiency of ECM

**DOI:** 10.3390/ma14092132

**Published:** 2021-04-22

**Authors:** Alexander Schupp, Oliver Beyss, Bob Rommes, Andreas Klink, Daniela Zander

**Affiliations:** 1 Chair of Corrosion and Corrosion Protection, Foundry Institute, RWTH Aachen University, Intzestr. 5, 52072 Aachen, Germany; a.schupp@gi.rwth-aachen.de (A.S.); o.beyss@gi.rwth-aachen.de (O.B.); 2 Laboratory for Machine Tools and Production Engineering (WZL), RWTH Aachen University, Campus-Boulevard 30, 52074 Aachen, Germany; b.rommes@wzl.rwth-aachen.de (B.R.); a.klink@wzl.rwth-aachen.de (A.K.)

**Keywords:** 42CrMo4 steel, electrochemical machining, rim zone modification, microstructure, process efficiency, transpassive dissolution, surface chemistry

## Abstract

The electrochemical machining (ECM) of 42CrMo4 steel in sodium nitrate solution is mechanistically characterized by transpassive material dissolution and the formation of a Fe_3−x_O_4_ mixed oxide at the surface. It is assumed that the efficiency of material removal during ECM depends on the structure and composition of this oxide layer as well as on the microstructure of the material. Therefore, 42CrMo4 in different microstructures (ferritic–pearlitic and martensitic) was subjected to two ECM processes with current densities of about 20 A/cm^2^ and 34 A/cm^2^, respectively. The composition of the process electrolyte was analyzed via mass spectrometry with inductively coupled plasma in order to obtain information on the efficiency of material removal and the reaction mechanisms. This was followed by an X-ray photoelectron spectroscopy analysis to detect the chemical composition and the binding states of chemical elements in the oxide formed during ECM. In summary, it has been demonstrated that the efficiency of material removal in both ECM processes is about 5–10% higher for martensitic 42CrMo4 than for ferritic–pearlitic 42CrMo4. This is on one hand attributed to the presence of the cementite phase at ferritic–pearlitic 42CrMo4, which promotes oxygen evolution and therefore has a negative effect on the material removal efficiency. On the other hand, it is assumed that an increasing proportion of Fe_2_O_3_ in the mixed oxide leads to an increase in the process efficiency.

## 1. Introduction

Electrochemical machining (ECM) belongs to the substractive manufacturing processes and is relevant to several industries, such as the aerospace industry [1,2,3], the machining of hard-to-cut materials [4,5,6] and medical technology [7]. The concept of ECM is based on the transpassive anodic dissolution of the material surface. The machining is performed by the application of an external voltage between the tool and the workpiece. This results in different electrochemical reactions influencing the efficiency of the process which is described by the charge yield.

Machining iron and steels, ECM is usually carried out in electrolytes with good electrical conductivity and a high salt content, such as sodium chloride or sodium nitrate solution. ECM processing in sodium chloride solution is characterized by a better process efficiency compared to sodium nitrate [8]. However, for low-alloyed steels such as the 42CrMo4 steel studied in this work, sodium chloride causes pitting, which has a negative effect on surface integrity [9]. Therefore, sodium nitrate solution is usually used for low-alloyed steels.

ECM processing of iron and steels in sodium nitrate solution is mechanistically a stationary two-step process at transpassive potentials typically obtained at DC voltages of 10–25 V and current densities of up to 200 A/cm^2^ [10]. The electrochemical reactions are determined by the transpassive formation of an oxide layer on the material surface and the subsequent dissolution of the oxide layer running parallel. Besides the anodic reactions also other reactions at the anode have to be considered as an additional influencing factor when considering the efficiency. The formation of oxygen consumes charges and therefore lowers the efficiency of ECM [11,12,13,14]. In general the electrochemical reactions are influenced on one hand by the machining parameters, the resulting local current densities and the electrolyte, and on the other hand by the surface chemistry as well as the rim zone composition and the microstructure.

The surface chemistry is determined by the composition and stoichiometry of the transpassively formed oxide layer during ECM and has a decisive impact on the efficiency of the anodic material removal and the surface quality that can be achieved for iron and steels by ECM [15,16]. According to Haisch et al. [17], the efficiency of material removal in the ECM process depends in particular on the electrical conductivity of the surface layers. Oxygen is preferentially generated on layers with good electrical conductivity, such as Fe_3_O_4_ [18], which has a negative effect on the efficiency of material removal; on poorly conductive layers, such as Fe_2_O_3_ [18] oxygen generation tends to be inhibited. For this reason, there have already been extensive studies on the formation and dissolution of the oxide layer in the ECM process. In 1984, Datta et al. [19] were able to demonstrate by atomic emission spectroscopy (AES) and X-ray photoelectron spectroscopy (XPS) that, at high transpassive current densities in sodium nitrate solution, an oxide layer is formed on iron which consists predominantly of Fe_2_O_3_. However, bivalent iron Fe (II) could also be detected in small amounts. In addition, Datta et al. [19] could prove that the thickness of the oxide layer is strongly dependent on the current density resulting from the ECM process. Up to a current density of 8 A/cm^2^, the thickness of the oxide layer increases with increasing current density and reaches a maximum at about 20 nm. As the current density increases further, the thickness of the oxide layer decreases again. The thickness of the oxide layer correlates with the amount of oxygen evolution. Below 8 A/cm^2^, oxygen evolution is the dominant reaction. With a further increase in current density, oxygen evolution decreases. Furthermore, Lohrengel et al. [11,12,13,14] were able to prove an additional influence of the current density on the stoichiometry of the oxide layer that was formed during electrochemical dissolution of pure iron in sodium nitrate solution. It was proposed that with increasing current density Fe_3_O_4_ is formed predominantly. The electrochemical investigations were performed in a micro flow cell and the oxidation states of iron in the process electrolyte were analyzed by UV-VIS. In contrast, Münninghoff [20] did not detect any influence of the current density on the stoichiometry of the transpassive oxide layer on iron using a comparable experimental setup to Lohrengel et al. [11,12,13,14].

However, Zander et al. [21] were able to demonstrate an influence of the current density on the composition of the ECM oxide layer for martensitic 42CrMo4. An increase in oxidation number of iron with increasing current density from predominantly Fe II to Fe III was observed by X-ray photoelectron spectroscopy. It was assumed that a mixed Fe_3-x_O_4_ layer forms on 42CrMo4 during the ECM process. This layer contains amounts of both Fe_2_O_3_ and Fe_3_O_4_, with the amount of Fe_2_O_3_ increasing with increasing current density which is in contrast to [11,12,13,14]. Furthermore, an enrichment of the oxide layer with chromium and molybdenum both for ECM processing at 20 A/cm^2^ and at 34 A/cm^2^ under industrial conditions was observed.

Besides the surface chemistry, the rim zone microstructure also has to be considered while studying the efficiency of ECM. It was observed that the microstructure, in particular the amount, size and shape of carbides, influences the ECM process of steels. Haisch et al. [22] showed that carbides behave electrochemically inert and are incorporated into the transpassively formed oxide layer during ECM of 100Cr6, C15, C45 and C60 steels in sodium nitrate and sodium chloride solution. Furthermore, the carbides showed a negative effect on the efficiency of ECM material removal. By comparing pearlitic and soft-annealed steels, Haisch et al. [22] were able to demonstrate that the coarser carbides in the pearlitic microstructure compared to the soft-annealed microstructure reduce the charge yield. This is mainly attributed to the preferential formation of oxygen on the electrically highly conductive carbides in the ECM process, which leads to current losses. Klocke et al. [23,24] and Harst [25] have carried out investigations on ECM of ferritic–pearlitic 42CrMo4. In contrast to Haisch et al. [8,22] they conclude that the cementite phase can also be dissolved electrochemically at high transpassive current densities. However, an enhanced selective dissolution of the ferrite phase compared to the cementite phase was also demonstrated by Klocke et al. [23,24] and Harst [25]. The investigations of Zander et al. [26] of ground, ferritic–pearlitic 42CrMo4 compared to EDM-processed (electrical discharge machining), partially austenitic 42CrMo4 revealed a lower material removal efficiency for the ground ferritic–pearlitic compared to the partially austenitic steel. This also was associated to the stronger oxygen reaction related to the cementite. In addition, it was observed that the influence of the microstructure on the efficiency was superimposed by roughness effects. However, there is still a lack in understanding the transpassive formation and composition of the oxide layer influencing the oxygen reaction. In addition, the microstructural impact on the oxygen evolution and the influence on the oxide layer has to be considered to evaluate the efficiency of the ECM of differently heat treated 42CrMo4. Therefore, this study focuses on the influence of surface chemistry and rim zone microstructure of 42CrMo4 on the efficiency of ECM.

42CrMo4 in martensitic and in ferritic–pearlitic microstructure was analyzed in a first step using electron microscopic examinations. Subsequently, the 42CrMo4 steel was subjected to two ECM processes with different current densities in both heat treatment states. During this process, the composition of the electrolyte was analyzed via mass spectrometry with inductively coupled plasma in order to obtain information on the efficiency of material removal and the reaction mechanisms taking place in the ECM process. This was followed by a comprehensive analysis of the oxide layers formed in the ECM process by X-ray photoelectron spectroscopy to detect differences in the chemical composition or in the binding states.

## 2. Materials and Methods

### 2.1. Materials

The main components of the heat-treatable steel 42CrMo4 (AISI4140) are iron, chromium, manganese, molybdenum and carbon. The chemical composition is summarized in Table 1.

ECM of 42CrMo4 was performed for two different microstructures: ferritic–pearlitic and martensitic. These microstructures were adjusted by heat treatment. The martensitic microstructure was achieved by quenching and tempering (QT). For this purpose, the material was austenitized in the furnace for 2 h at 850 °C and then quenched to 60 °C in oil. The steel was then tempered for another 4 h at 400 °C and then cooled in air to room temperature. Additionally, for the ferritic–pearlitic (FP) microstructure, the 42CrMo4 was first austenitized for 2 h at 850 °C in a vacuum furnace. Subsequently, the steel was cooled to room temperature in the furnace at a rate of 1.7 K/min. After heat treatment, the material was cut into plates with an edge length of 10 mm × 10 mm and a height of 2 mm.

### 2.2. The Experimental Test of ECM

**Figure 1:**
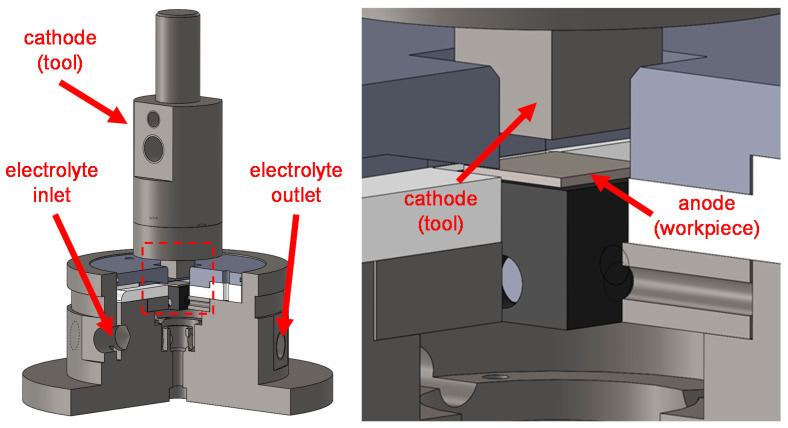
Experimental setup for the electrochemical machining.

Both the ferritic–pearlitic and the quenched and tempered (martensitic) 42CrMo4 were subjected to ECM using an EMAG PTS 1500 (EMAG GmbH & Co. KG, Salach, Germany). An external DC voltage of 19 V was applied between workpiece (anode, A = 1 cm^2^) and tool (cathode, A = 1 cm^2^). Sodium nitrate electrolyte (conductivity: 135 mS/cm; pH 8; temperature: 36 °C; flow rate: 4.4 L/min, Carl Roth GmbH & Co. KG, Karlsruhe, Germany) was used as process electrolyte. The experimental setup is shown in Figure 1.

On both ferritic–pearlitic and martensitic 42CrMo4, two different ECM processes were performed: ECM-A and ECM-B. These processes differ mainly in working gap and cathode feed rate, as shown in Table 2. In addition, the machining time of ECM-A is three times longer than ECM-B. The process parameters were selected on the basis of own preliminary experiments. Care was taken to ensure that no flow grooves occur during processing and that the influence of electrolyte hydrodynamics on the process could be neglected for both ECM-A and ECM-B [16,25]. In addition, the width of the working gap should be almost constant during the process to ensure constant current density.

The current flow and the height reduction of the samples are measured during the ECM process. This allows statements on the efficiency of the anodic material removal. In the context of this work, efficiency describes how efficiently the applied current is used to dissolve the material and how much current is lost in the process. Therefore, the current flow was converted into an idealized, loss-free mass removal via Faraday’s law, while the actual measured height reduction provides information about the real material removal. The quotient of the real and the idealized material removal is thereby defined as the charge yield η. η is thus an important measure of the efficiency of material removal. The calculation of η and the idealized mass removal via Faraday’s law has already been described in a previous publication in more detail [26].

### 2.3. Chemical Analysis of the Electrolyte before and after ECM

In order to determine the content of the dissolved elements iron, chromium, manganese and molybdenum in the sodium nitrate electrolyte before as well as after the ECM process, a mass spectrometer with inductively coupled plasma (ICP-MS) was applied. The NexION 2000 mass spectrometer (PerkinElmer LAS (Germany) GmbH, Rodgau, Germany) was used for this purpose. The mass content of the elements in the process electrolyte provides information about the real material removal as well as about the reaction mechanisms during the ECM process. Electrolyte samples were taken continuously throughout the ECM process. Only in the first 10 s and the last 10 s of the process no electrolyte samples were taken. This implies that electrolyte samples were taken for a period of 80 s in the ECM-A process, and for a period of about 10 s in the ECM-B process. At least four samples each from the ECM-A and ECM-B processes were then diluted with 2% nitric acid in a ratio of 1 to 50 and then analyzed by ICP-MS.

### 2.4. Scanning Electron Microscopy and Electron Backscatter Diffraction

To obtain information on the microstructure, cross-sections of the martensitic and the ferritic–pearlitic 42CrMo4 were prepared. Before scanning electron microscopy (SEM) analysis in a Zeiss Ultra 55 (Carl Zeiss Microscopy Deutschland GmbH, Oberkochen, Germany), the cross-sections were subjected to an etchant solution made of 2 g CuCl_2_, 40 mL ethanol and 40 mL concentrated hydrochloric acid for 10 s at room temperature. In addition, electron backscatter diffraction (EBSD) images of polished cross sections were recorded.

After electrochemical machining, top view images of the surfaces were taken by a Zeiss Supra 55 VP scanning electron microscope (Carl Zeiss Microscopy Deutschland GmbH, Oberkochen, Germany).

### 2.5. Rim Zone Analysis of the Native Oxide and the Oxide after ECM Processing

X-ray photoelectron spectroscopy (XPS, Kratos Analytical Ltd, Manchester, UK) provides information about the chemical composition of the studied rim as well as about the bonding states of the elements. XPS analyses of the ground, ECM-A and ECM-B surfaces were performed in a Kratos Axis Supra instrument. A monochromatic Al Kα X-ray source (*hν* = 1486.6 eV) has been used to obtain high resolution spectra of the O 1s, C 1s, Fe 2p, Cr 2p, Mo 3d, and N 1s regions. The XPS measurements were carried out using a pass energy of 20 eV and a step size of 0.1 eV. The ESCApe software (Kratos Analytical Ltd., Manchester, UK) was used to perform peak deconvolution and quantification. Charge correction of all spectra was conducted by assigning the adventitious C-C contamination component in the C 1s peak to 284.8 eV. Peak fitting was performed using Shirley type backgrounds and Gauss-Lorentz (30/70) type line shapes for most of the components. Only for metallic Fe, asymmetric line shapes were used.

## 3. Results

### 3.1. Microstructure

**Figure 2:**
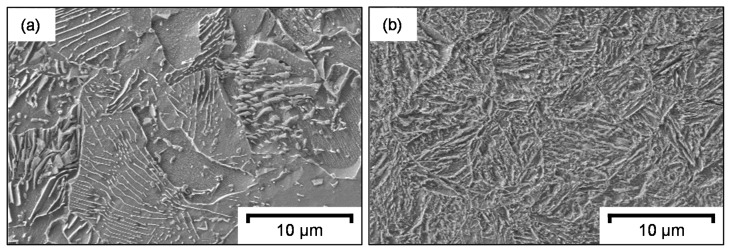
CuCl_2_ etching of (**a**) ferritic–pearlitic and (**b**) martensitic 42CrMo4 steel.

The microstructure of ferritic–pearlitic 42CrMo4 differs from martensitic 42CrMo4. The differences were made visible by CuCl_2_ etching, as demonstrated in Figure 2. In the ferritic–pearlitic microstructure, the pearlite grains with the protruding cementite lamellae can be easily distinguished from the cementite-free ferrite grains. The martensitic microstructure instead consists of elongated martensite lancets.

**Figure 3:**
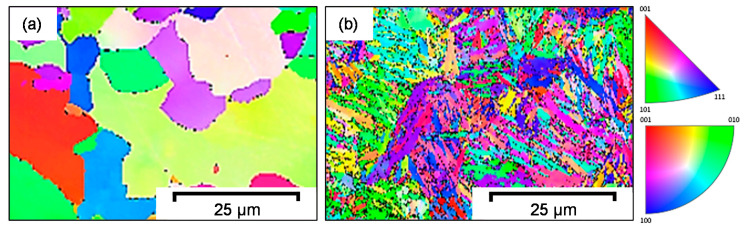
EBSD images of (**a**) ferritic–pearlitic and (**b**) martensitic 42CrMo4 steel.

EBSD images additionally show that the martensite grains are not entirely disordered. As demonstrated in Figure 3b, the individual grains are present in clusters with similar crystallographic orientation. These clusters are assumed to be former austenite grains that were present in the material prior to quenching during heat treatment. In the case of the ferritic–pearlitic 42CrMo4 Figure 3a, no representation of the cementite lamellae was possible due to the small size of the lamellae.

The differences of the microstructure were also obvious in the mechanical properties of the material. For example, the hardness of the ferritic–pearlitic 42CrMo4 is 190 ± 10 HV0.2, while the hardness of the martensitic 42CrMo4 is 470 ± 10 HV0.2.

### 3.2. Electrochemical Machining

According to Table 2, two different ECM processes were carried out on martensitic and ferritic–pearlitic 42CrMo4. For the ECM-A process, the average current was almost constant 20 A, and for ECM-B 34 A. The final working gap was about 1.1 mm for ECM-A and 0.5 mm for ECM-B. Therefore, a nearly stationary process is assumed for both ECM-A and ECM-B after a short transition phase of less than 5 s.

To obtain the material removal rate of each process, three different calculations were performed: The current densities were used for the calculation with Faraday’s law and resulted in an idealized removal rate. A real material removal was calculated via the measurement of the sample height as well as via ICP-MS measurements of the element composition in the sodium nitrate electrolyte. For the ferritic–pearlitic steel, mass losses of 300 mg for ECM-A and 205 mg for ECM-B were determined by ICP-MS; for the martensitic steel, the mass loss is 360 mg for ECM-A and 230 mg for ECM-B. Table 3 shows a higher idealized removal rate compared to the real removal rate due to the assumption of a 100 % charge yield for the idealized conditions. In addition, differences between the two different calculation approaches for the real material removal rate were obtained. In general, the removal rate calculated from the overall dissolved alloying element content in the electrolyte is higher compared to the one calculated by height measurements. This difference is assumed to relate to the known non-uniform material removal along the surface of the component during ECM. Since only the highest point of the surface is determined with the height measurement, the material removal is underestimated with this measurement method. For this reason, the calculation of the material removal via ICP-MS is more suitable and used for the following calculations.

**Figure 4:**
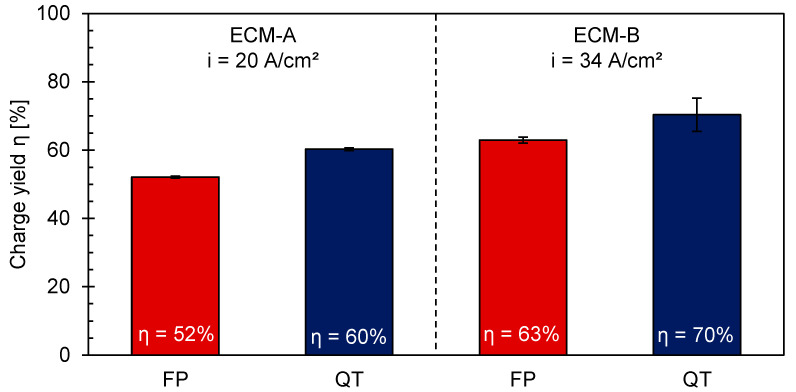
Process efficiency of ECM-A and ECM-B for ferritic–pearlitic (FP) and martensitic (QT) [21] 42CrMo4.

Comparing the real removal rates of the different microstructures, Table 3 illustrates a lower real material removal rate for the ferritic–pearlitic 42CrMo4 than for to the martensitic 42CrMo4. This tendency is also reflected for the efficiency calculation of η for the individual processes (Figure 4). For the ECM-A process, the charge yield of FP is 52%, while for QT it is 60%. For the ECM-B process, the charge yield is in general higher for both microstructures compared to ECM-A. Still a charge yield difference between FP and QT of about 7% was observed.

**Figure 5:**
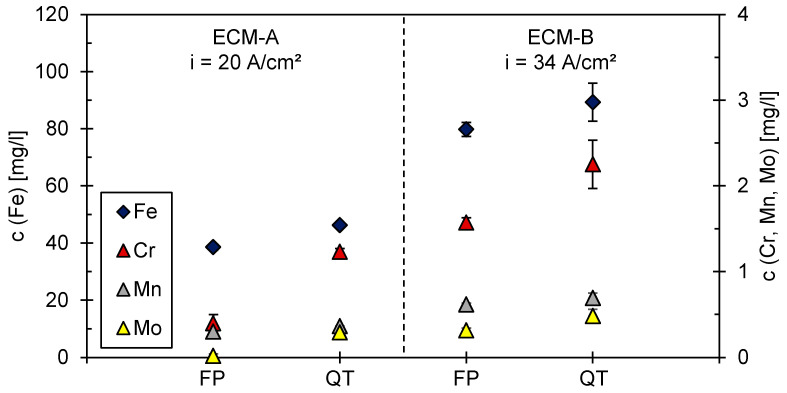
ICP-MS analysis of the process electrolyte after ECM-A and ECM-B for ferritic–pearlitic (FP) and martensitic (QT) [21] 42CrMo4.

The results of the ICP-MS measurements are not only required for the calculation of the charge yield η, but also provide information on whether single alloying elements are transferred into solution in an enhanced or attenuated manner compared to iron during the ECM process. Figure 5 reveals that the element chromium is more strongly dissolved relative to iron in the martensitic 42CrMo4 compared to ferritic–pearlitic 42CrMo4 in both the ECM-A and ECM-B process. A similar trend seems to be present for molybdenum. However, no definitive statement can be made for the FP ECM-A measurement, as the concentration of molybdenum in the electrolyte is close to the limit of detectability. No indication of a preferential dissolution of manganese with respect to iron could be found for all four processes.

### 3.3. Surface Analysis after ECM

**Figure 6:**
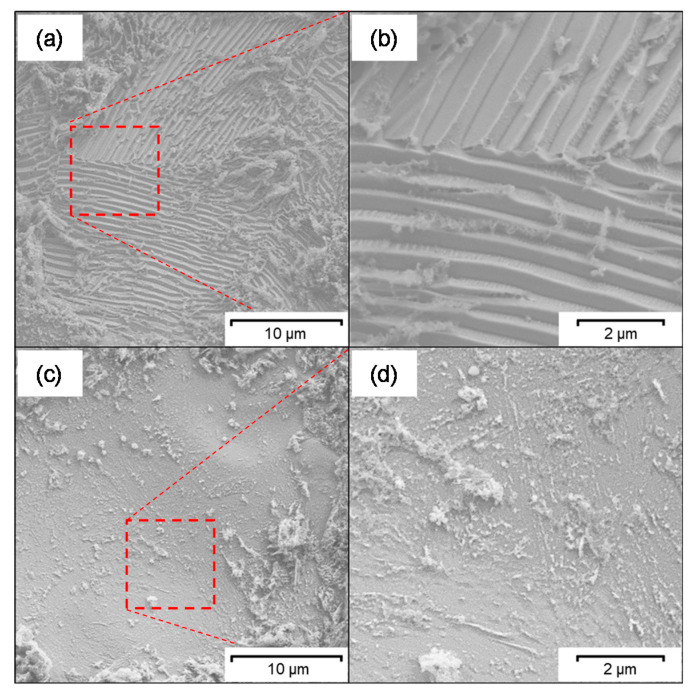
SEM images of (**a**,**b**) ferritic–pearlitic and (**c**,**d**) martensitic 42CrMo4 after ECM-B.

The influence of the ferritic–pearlitic and martensitic microstructure as well as of the surface chemistry on the efficiency of ECM material removal was studied by SEM and XPS. SEM (Figure 6) shows a relief-like morphology in some areas of the surface exemplary after ECM-B (Figure 6a,b). This is most likely due to pearlite grains in which selective material removal has taken place between the ferrite and cementite lamellae. No such relief-like surface areas have been detected in the martensitic 42CrMo4. Both microstructures exhibit no preferred dissolution along grain boundaries.

XPS was used to analyze the chemical composition of the rim zone (information depth about 10 nm) both in the ground state before ECM and after the ECM-A and ECM-B process. As Table 4 illustrates, the surface compositions of the two investigated microstructures are similar to each other within one process. However, higher iron and lower chromium content was observed for ground compared to ECM processed 42CrMo4. Furthermore, ECM processing leads to a significant increase in the oxygen content in the rim zone with a simultaneous decrease in the iron content. In addition, there is an enrichment of molybdenum in the rim zone after ECM. The elements nitrogen and manganese could be detected only qualitatively. However, no meaningful quantitative analysis was possible for these elements due to the weakness of the measurement signal. This is due to the locally different roughness of the surfaces that were examined.

**Figure 7:**
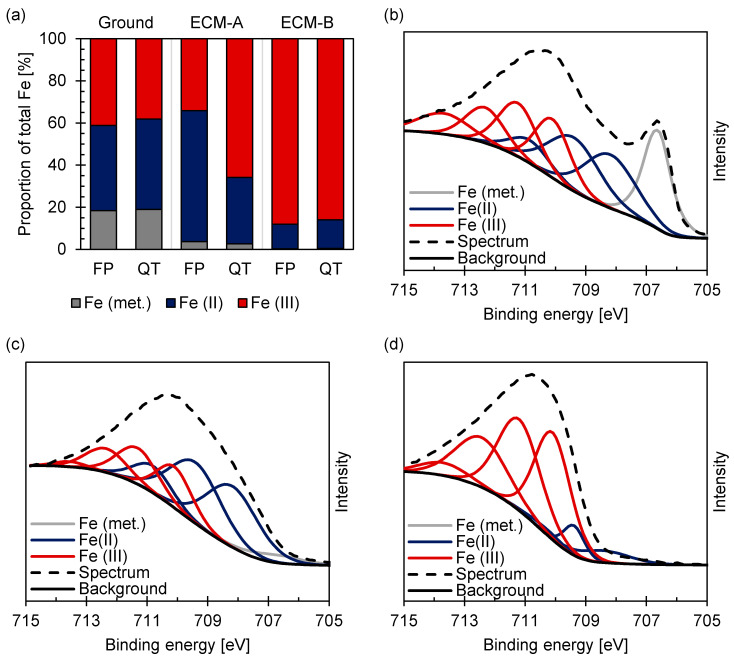
Proportion of oxidation states of iron for (**a**) ferritic–pearlitic and martensitic [21] 42CrMo4 and XPS spectra of the Fe2p3/2 region for (**b**) ground, (**c**) ECM-A and (**d**) ECM-B ferritic–pearlitic 42CrMo4 steel fitted according to [28,29].

In addition to the chemical composition, the XPS measurements also provide information on the binding state of the elements. Figure 7 reveals that in the case of ferritic–pearlitic 42CrMo4 in the ground state, the element iron is present in metallic form to about 20% and as Fe (II) and Fe (III) to about 40% each indicating the formation of a mixed iron oxide Fe_3-x_O_4_. After ECM-A processing, the metallic content is only about 3%, while the Fe (II) content increases significantly up to about 60%. After ECM-B processing, Fe (III) becomes the dominant state with a content of about 90%.

Comparing ground ferritic–pearlitic with martensitic [21] 42CrMo4 no changes of the proportion of metallic Fe, Fe (II) and Fe (III) were observed (Figure 7a), whereas significant differences in the proportions of Fe (II) and Fe (III) occurred for the different microstructures after ECM-A. A ratio of Fe (III)/Fe (II) = 1/2 was determined for ferritic–pearlitic 42CrMo4. Contrastingly, a ratio of Fe (III)/Fe (II) = 2/1 was observed for the martensitic microstructure. Furthermore, no differences in the proportion of the iron species between the two microstructures were observed after ECM-B, but a general increase in the ratio of Fe (III)/Fe (II) compared to the ground and ECM-A state.

**Figure 8:**
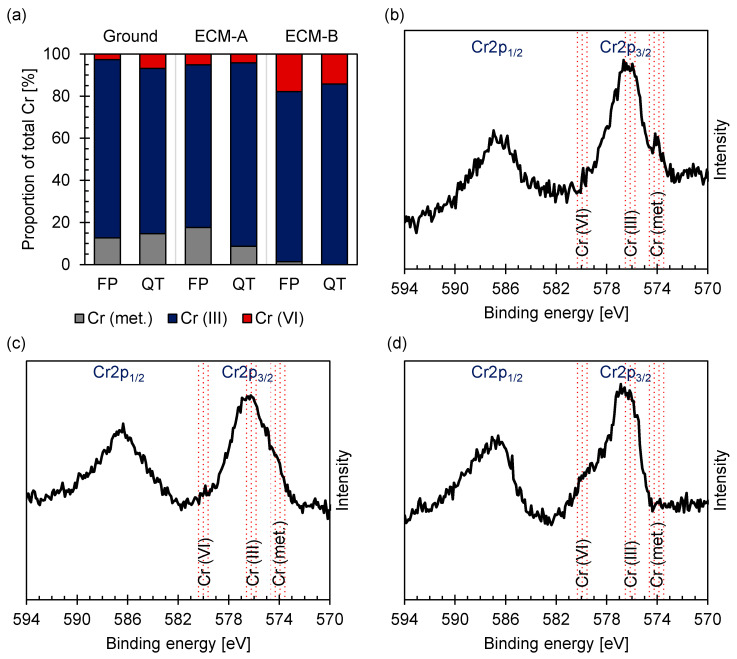
Proportion of oxidation states of chromium for (**a**) ferritic–pearlitic and martensitic [21] 42CrMo4 and XPS spectra of the Cr2p region for (**b**) ground, (**c**) ECM-A and (**d**) ECM-B ferritic–pearlitic 42CrMo4 steel fitted according to [29].

The analysis of the XPS spectrum of the Cr2p region (Figure 8) according to Biesinger et al. [29] revealed for the ferritic–pearlitic 42CrMo4 that metallic chromium is present in a proportion of 10–20% both in the ground state and in the ECM-A state. The majority of the chromium is present as Cr (III). However, almost no metallic chromium was detected on the surface but about 20% Cr (VI) and 80% Cr (III) after the ECM-B process. These results for ferritic–pearlitic 42CrMo4 steel are almost identical to those for martensitic 42CrMo4 [21]. Only an increased content of metallic chromium in FP ECM-A compared to QT ECM-A is notable.

**Figure 9:**
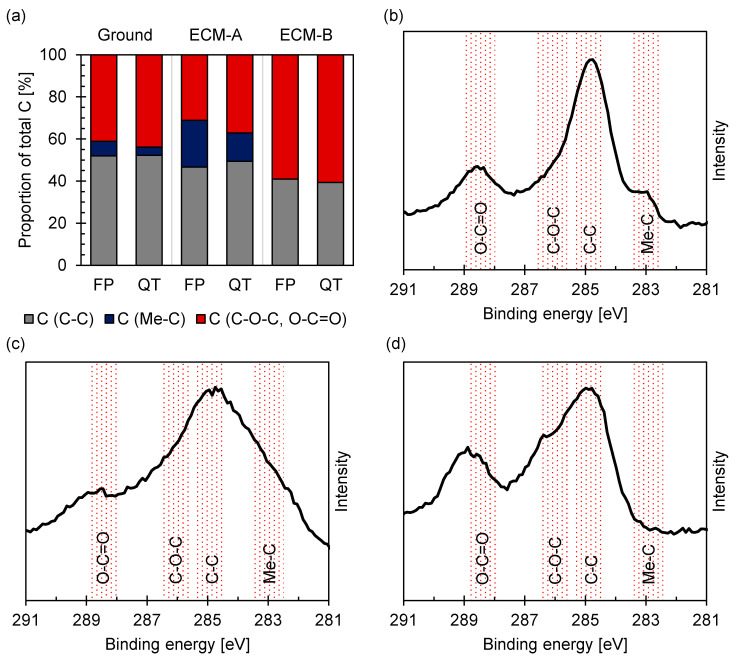
Proportion of binding states of carbon for (**a**) ferritic–pearlitic and martensitic 42CrMo4 and XPS spectra of the C1s region for (**b**) ground, (**c**) ECM-A and (**d**) ECM-B ferritic–pearlitic 42CrMo4 steel fitted according to [30].

Carbon, which was detected on all surfaces by XPS, was associated predominantly to organic contaminants, as shown in Figure 9. In the case of the ferritic–pearlitic 42CrMo4, a higher amount of carbides was also detected on both the ground and ECM-A surfaces compared to martensitic 42CrMo4. The proportion of carbides to total carbon is about 10% for the ground surface and about 20% for the ECM-A surface. In contrast, no carbides were detected at the ferritic–pearlitic ECM-B surface for both microstructures.

## 4. Discussion

Within the scope of the present work, the influence of the transpassive oxide layer formation and chemistry as well as the microstructure and its effect on the mixed oxide of heat treated 42CrMo4 on the efficiency of ECM was investigated. First, the efficiency of the ECM process was determined. Second, an analysis of the electrolyte was carried out and third, a comprehensive characterization of the ECM oxide layer and the local microstructure after ECM was performed.

It has been demonstrated that the efficiency for both the martensitic and the ferritic-pearlitic 42CrMo4 is higher for ECM-B than for ECM-A (Figure 4). This is believed to relate to the formation of the Fe_3-x_O_4_ mixed oxide and the observed change of the stoichiometry of the mixed oxide with increasing current density (Figure 7) during ECM of ferritic–pearlitic and martensitic 42CrMo4. In both the ferritic–pearlitic and the martensitic 42CrMo4, an oxide consisting predominantly of Fe_2_O_3_ is present in the ECM-B process at high current densities. This oxide is expected to exhibit a lower electrical conductivity than the predominantly oxide Fe_3_O_4_ formed during the ECM-A process with lower current densities [18]. According to Haisch et al. [17], oxygen evolution has a negative effect on the efficiency of material removal and occurs preferentially on layers with good electrical conductivity. Therefore, it is assumed that oxygen evolution is more inhibited during the ECM-B process than during ECM-A. This results in lower charge losses due to oxygen evolution and an improved charge yield in the ECM-B process.

The generally increased efficiency of material removal with increasing current density for ECM-B compared to ECM-A for both microstructures can be partially explained by the different composition and stoichiometry of the oxide layer. Furthermore, the lower efficiency of material removal of the ferritic–pearlitic state compared to the martensitic state in ECM-A is also associated to the differences in the composition and stoichiometry of the mixed oxide layer. However, for the ECM-B process it was observed, that the composition of the mixed oxide layers are almost identical for the ferritic–pearlitic and the martensitic 42CrMo4. Since also an increased charge yield of the martensitic 42CrMo4 in ECM-B was obtained, at least one more influencing factor besides the formation and dissolution of the mixed oxide layer is assumed to affect the efficiency in the ECM process.

For this reason, an influence of the microstructure on the efficiency of the ECM besides the influence of the mixed oxide layer was considered. As shown in Figure 4, the efficiency of material removal is higher for the martensitic 42CrMo4 than for the ferritic–pearlitic 42CrMo4 in both the ECM-A and ECM-B process. It is assumed that the carbides within the pearlite phase are responsible for the difference in charge yield. This assumption is supported by studies of Haisch et al. [22] on the electrochemical dissolution of steels. It was reported that during electrochemical dissolution of the material, the carbides in the microstructure behave largely inert and accumulate at the surface layer. The lamellar carbides are strongly embedded in the underneath lying pearlitic structure and cannot be removed easily. At the surface, the accumulated carbides hinder the further dissolution of the underneath pearlitic microstructure. Furthermore, due to their good electrical conductivity, they are also a preferred site for the formation of oxygen. This lowers the charge yield of the electrochemical material dissolution and results in a decreasing charge yield [8,17,22].

An accumulation of lamellar carbides on ferritic–pearlitic 42CrMo4 during ECM in NaNO_3_ solution was revealed by SEM (Figure 6) and confirmed by Ehle et al. [31] by transmission electron microscopy as well as by Klocke et al. [23,24] and Harst [25]. It is assumed that the increased carbide density at the surface of the ferritic–pearlitic 42CrMo4 leads to a lower charge yield compared to the carbide-poor, martensitic 42CrMo4 due to an increased oxygen evolution. In contrast to Haisch et al. [8,17,22] it is considered that the cementite phase is not electrochemically inert, but dissolves significantly slower than the ferritic phase [23,24,25,26]. Taking into account that the XPS measurements (Figure 9) showed no enrichment of the rim zone with carbides for ECM-B, also the thickness of the mixed oxide layer has to be considered. If carbon is predominantly enriched at the metal/oxide interface, it would consequently be more difficult to detect carbon buried under a thick oxide layer by XPS. Information about the oxide layer thickness could be provided by an XPS depth profile generated by sputtering. However, due to the high surface roughness, the depth information obtained from this is limited.

In addition to the influence of the cementite phase, a microstructural influence of the grain orientation on the efficiency of material removal was also considered. For example, Schreiber et al. [32] were able to demonstrate on iron that a different grain orientation can lead to locally different anodic dissolution. However, in the ECM processing of 42CrMo4 in sodium nitrate solution described in the present work, no preferential dissolution of individual grains based on their orientation was determined (Figure 6).

Summarizing, a strong influence of surface chemistry and rim zone microstructure of 42CrMo4 on the efficiency of ECM was proven. This is especially due to the interaction of the two influencing factors. This can be observed especially in ECM-A, where the efficiency difference between the ferritic–pearlitic and the martensitic 42CrMo4 can be caused by both the different microstructure and local surface chemistry which is controlled by the formation of a Fe_3-x_O_4_ mixed oxide.

## 5. Conclusions

42CrMo4 was electrochemically processed in sodium nitrate solution in two microstructures-ferritic–pearlitic and martensitic-at a current density of about 20 A/cm^2^ and 34 A/cm^2^, respectively. In each case, the efficiency of material removal was determined by ICP-MS measurement of the process electrolyte. The efficiency was then correlated with the microstructure of the material which was analyzed by SEM and the surface chemistry which was analyzed by XPS. The following conclusions were made:

- An increase in the current density in the ECM process from about 20 to 34 A/cm^2^ leads to a change of the composition and stoichiometry of the transpassively formed Fe_3-x_O_4_ mixed oxide layer on the material surface. The proportion of Fe_2_O_3_ in the oxide layer increases with increasing current density. Simultaneously, an increase in process efficiency can also be observed. This is partially attributed to the local composition of the mixed oxide. Fe_2_O_3_ which preferentially forms at high current densities is electrically less conductive than Fe_3_O_4_ forming at low current densities. Since the charge-consuming oxygen evolution occurs preferentially on surfaces with good electrical conductivity, it is assumed that oxygen evolution tends to decrease with increasing Fe_2_O_3_ content in the rim zone. This has a positive effect on the efficiency of the ECM process.
- The microstructure of the material exhibits a significant influence on the efficiency of material removal. The efficiency of material removal of martensitic 42CrMo4 is about 10% higher compared to ferritic–pearlitic 42CrMo4. This is predominantly attributed to the presence of cementite lamellae in the ferritic–pearlitic microstructure, which accumulate on the surface during the ECM by selective dissolution of the ferritic phase. The undesirable reaction of oxygen evolution takes place preferentially on cementite, which has a negative effect on the charge yield and thus the efficiency of the process.

Based on the observed influence of the surface chemistry and the microstructure on the ECM process, further work is underway to investigate whether surface properties (e.g., oxide layer stoichiometry, chemical composition, phase distribution, roughness) can be targeted by the ECM process and thereby improve the functionality of the surfaces. Possible functional properties that will be addressed include resistance to aqueous corrosion or high-temperature resistance. In addition, there are considerations whether an improvement of the adhesion of coatings applied to the surface is possible by a specific adjustment of the surface chemistry through a suitable ECM process design.

## Figures and Tables

**Table 1:** Chemical composition of 42CrMo4 [27].

Element	wt.%	at.%
C	0.43	1.96
Si	0.26	0.51
Mn	0.74	0.74
P	0.01	0.018
S	<0.001	<0.002
Cr	1.09	1.15
Mo	0.25	0.14
Fe	Bal.	Bal.

**Table 2:** ECM process parameters.

Process	Initial Working Gap [µm]	Feed Rate Cathode [mm/min]	Machining Time [s]
ECM-A	1000	0.1	100
ECM-B	500	0.6	33.3

**Table 3:** Average material removal rates for ferritic–pearlitic (FP) and martensitic (QT) [21] 42CrMo4 during ECM-A and ECM-B.

Process	Removal Rate Calculated by Height Measurement [mg/(s *cm^2^)]	Removal Rate Calculated from Electrolyte by ICP-MS [mg/(s *cm^2^)]	Idealized Removal Rate Calculated by Faraday’s Law [mg/(s *cm^2^)]
FP ECM-A	2.4 ± 0.1	3.0 ± 0.1	5.8 ± 0.1
QT ECM-A [21]	2.8 ± 0.4	3.6 ± 0.1	5.9 ± 0.1
FP ECM-B	4.9 ± 0.8	6.2 ± 0.2	9.8 ± 0.2
QT ECM-B [21]	5.3 ± 1.1	7.0 ± 0.5	9.8 ± 0.1

**Table 4:** Chemical composition of the rim zone of ferritic–pearlitic (FP) and martensitic (QT) 42CrMo4 in ground, ECM-A and ECM-B state.

Surface	Fe [wt.%]	Cr [wt.%]	Mo [wt.%]	O [wt.%]	C [wt.%]
FP ground	55.0	1.5	0.4	32.1	11.0
QT ground	51.9	1.0	0.3	31.4	15.5
FP ECM-A	44.0	2.3	0.5	45.1	8.0
QT ECM-A	47.4	2.6	0.4	42.9	6.6
FP ECM-B	45.2	2.1	0.6	46.8	5.4
QT ECM-B	43.6	2.5	0.4	46.7	6.8

## Data Availability

The data presented in this study are available on request from the corresponding author.

## References

[1] Yang Y., Zhu D., Lu J. (2020). Surface Morphology Analysis of 1Cr11Ni2W2MoV Steel Subjected to Electrochemical Machining in NaNO_3_ Solution. J. Electrochem. Soc..

[2] Klocke F., Zeis M., Klink A. (2015). Interdisciplinary modelling of the electrochemical machining process for engine blades. CIRP Ann..

[3] Zhao Z.Q., Ma Z.G., Liu Y.T., Wu X.L., Guo H. (2018). Optimization and Affections of Cathode Feed Angle on Machining Accuracy Error Distribution of Aero-Engine Blade in Electrochemical Machining. Key Eng. Mater..

[4] Tang Y., Xu Z. (2018). The electrochemical dissolution characteristics of GH4169 nickel base super alloy in the condition of electrochemical machining. Int. J. Electrochem. Sci..

[5] Wang D., He B., Cao W. (2019). Enhancement of the localization effect during electrochemical machining of inconel 718 by using an alkaline solution. Appl. Sci..

[6] Schubert N., Schneider M., Michaelis A., Manko M., Lohrengel M.M. (2018). Electrochemical machining of tungsten carbide. J. Solid. State. Electrochem..

[7] Kamaraj A.B., Sundaram M.M., Mathew R. (2013). Ultra high aspect ratio penetrating metal microelectrodes for biomedical applications. Microsyst. Technol..

[8] Haisch T., Mittemeijer E., Schultze J.W. (2001). Electrochemical machining of the steel 100Cr6 in aqueous NaCl and NaNO3 solutions: Microstructure of surface films formed by carbides. Electrochim. Acta.

[9] Klocke F., Harst S., Ehle L., Zeis M., Klink A. (2018). Surface integrity in electrochemical machining processes: An analysis on material modifications occurring during electrochemical machining. Proc. Inst. Mech. Eng. Part B J. Eng. Manuf..

[10] Rajurkar K.P., Sundaram M.M., Malshe A.P. (2013). Review of electrochemical and electrodischarge machining. Procedia Cirp..

[11] Lohrengel M.M., Klüppel I., Rosenkranz C., Bettermann H., Schultze J.W. (2003). Microscopic investigations of electrochemical machining of Fe in NaNO_3_. Electrochim. Acta.

[12] Lohrengel M.M. (2005). Pulsed electrochemical machining of iron in NaNO_3_: Fundamentals and new aspects. Mater. Manuf. Process..

[13] Lohrengel M.M., Rosenkranz C. (2005). Microelectrochemical surface and product investigations during electrochemical machining (ECM) in NaNO_3_. Corros. Sci..

[14] Lohrengel M.M., Rosenkranz C., Rohrbeck D. (2006). The iron/electrolyte interface at extremely large current densities. Microchim. Acta.

[15] Hoare J.P. (1970). Oxide film studies on iron in electrochemical machining electrolytes. J. Electrochem. Soc..

[16] Bergs T., Harst S. (2020). Development of a Process Signature for Electrochemical Machining. CIRP Ann..

[17] Haisch T., Mittemeijer E.J., Schultze J.W. (2004). High rate anodic dissolution of 100Cr6 steel in aqueous NaNO_3_ solution. J. Appl. Electrochem..

[18] Kaesche H. (1990). Die Korrosion der Metalle. Physikalisch-Chemische Prinzipien und Aktuelle Probleme.

[19] Datta M., Mathieu H.J., Landolt D. (1984). AES/XPS study of transpassive films on iron in nitrate solution. J. Electrochem. Soc..

[20] Münninghoff T.R. (2012). Mechanismen der anodischen Auflösung von Metallen und Legierungen bei extrem hohen Stromdichten. Doctoral Dissertation.

[21] Zander D., Schupp A., Beyss O., Rommes B., Klink A. (2021). Oxide Formation during Transpassive Material Removal of Martensitic 42CrMo4 Steel by Electrochemical Machining. Materials.

[22] Haisch T., Mittemeijer E.J., Schultze J.W. (2002). On the influence of microstructure and carbide content of steels on the electrochemical dissolution process in aqueous NaCl-electrolytes. Mater. Corros..

[23] Klocke F., Harst S., Karges F., Zeis M., Klink A. (2017). Modeling of the electrochemical dissolution process for a two-phase material in a passivating electrolyte system. Procedia CIRP.

[24] Klocke F., Harst S., Zeis M., Klink A. (2018). Modeling and simulation of the microstructure evolution of 42CrMo4 steel during electrochemical machining. Procedia CIRP.

[25] Harst S. (2019). Entwicklung einer Prozesssignatur für die Elektrochemische Metallbearbeitung. Doctoral Dissertation.

[26] Zander D., Schupp A., Mergenthaler S., Pütz R.D., Altenbach C. (2021). Impact of rim zone modifications on the surface finishing of ferritic-pearlitic 42CrMo4 using electrochemical machining. Mater. Corros..

[27] Zander D., Klink A., Harst S., Klocke F., Altenbach C. (2019). Influence of machining processes on rim zone properties and high temperature oxidation behavior of 42CrMo4. Mater. Corros..

[28] Grosvenor A.P., Kobe B.A., Biesinger M.C., McIntyre N.S. (2004). Investigation of multiplet splitting of Fe 2p XPS spectra and bonding in iron compounds. Surf. Interface Anal..

[29] Biesinger M.C., Payne B.P., Grosvenor A.P., Lau L.W., Gerson A.R., Smart R.S.C. (2011). Resolving surface chemical states in XPS analysis of first row transition metals, oxides and hydroxides: Cr, Mn, Fe, Co and Ni. Appl. Surf. Sci..

[30] XPS Simplified. https://xpssimplified.com/elements/carbon.php.

[31] Ehle L., Harst S., Meyer H., Schupp A., Beyss O., Rommes B., Klink A., Schwedt A., Zander D., Weirich T.E. Microstructural and chemical surface and rim zone changes of ferrite perlite 42CrMo4 steel after electrochemical machining. Materialwiss. Werkstofftech..

[32] Schreiber A., Rosenkranz C., Lohrengel M.M. (2007). Grain-dependent anodic dissolution of iron. Electrochim. Acta.

